# Improved triplex real‐time PCR with endogenous control for synchronous identification of DNA from chicken, duck, and goose meat

**DOI:** 10.1002/fsn3.2272

**Published:** 2021-04-02

**Authors:** Guo‐Qiang Liu, Jian‐Xing Luo, Wei‐Liang Xu, Chun‐Dong Li, Yuan‐Sheng Guo, Liang Guo

**Affiliations:** ^1^ Xilin Gol Food Testing and Risk Assessment Center Xilin Gol Institute of Bioengineering Xilingol Vocational College Xilinhot China

**Keywords:** authentication, endogenous control, poultry, triplex real‐time PCR

## Abstract

The authentication and labeling of meat products, concerning origins and species, are key to fair trade and to protect consumer interests in the market. We developed an improved triplex real‐time PCR approach to simultaneously identify chicken, duck, and goose DNA in meat, including an endogenous control to avoid false negatives. Our method specifically detected DNA from chicken, duck, and goose, and showed no cross‐reaction with DNA extracted from other meat types. The detection limits of chicken, duck, and goose DNA were 0.001–0.00025 ng, 0.0025–0.0001 ng, and 0.001–0.00001 ng, respectively, and we were able to simultaneously identify DNA from two distinct origins using as little as 0.1% of total meat weight. Our newly generated triplex real‐time PCR method with endogenous control exhibited high specificity, sensitivity, and efficiency for simultaneous identification of DNA from chicken, duck, and goose in meat.

## Graphical abstract

This study is the first triplex real‐time PCR with an endogenous control for synchronous identification of DNA from chicken, duck, and goose meat. In view of the evaluation indicators, the designed technique is specific, sensitive, and efficient.

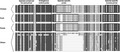

## 1 INTRODUCTION

In the last 50 years, poultry production has increased at global scale; the rate of increase in number is over twice that in human population (Bazer et al., 2020). Poultry is a source of high‐quality animal proteins and is often used as an ingredient in meat products. In addition, meat alteration with lower‐cost products, such as chicken, duck, or goose meat, has been used to illegitimately achieve higher financial profits. These fraudulent practices of adulteration might affect fair competition and do not consider the consumer interests in the market (Abbas et al., 2018; Bohme et al., 2019). Regulations have been applied to impose restrictions on the adulteration of meat products. To implement these legislations, there is an increasing demand for robust methods to analyze the authenticity of meat species claimed by manufacturers or distributors. Therefore, a specific, sensitive, and efficient method for the authentication of poultry meat (chicken, duck, and goose) is essential to the supervision of market practices.

At present, DNA‐based methods have been used to authenticate species (Rahmati et al., 2016; Xu et al., 2013). Poultry authentication in meat has been mainly focused on chicken, duck, and goose identification (Amaral et al., 2015; Furutani et al., 2017; Hou et al., 2015; Kesmen et al., 2012; Martin et al., 2007; Pegels et al., 2012; Thanakiatkrai et al., 2019). Conventional PCR (Amaral et al., 2015; Martin et al., 2007; Yao et al., 2020), multiplex PCR (Hou et al., 2015; Thanakiatkrai et al., 2019), and real‐time PCR (Furutani et al., 2017; Kesmen et al., 2012; Pegels et al., 2012) are highly specific and efficient methods which have been widely adopted in the detection of meat adulteration. *Taq*Man real‐time PCR, based on probes labeled by different fluorescent reporters, combines the advantages of multiplex and real‐time PCR (Guo et al., 2018, 2020; Iwobi et al., 2015; Köppel et al., 2013; Xu et al., 2018). In addition, the inclusion of endogenous control reflects authentically the normal amplification reaction and decreases the occurrence of false negative results (Bacich et al., 2011; Guo et al., 2018, 2020; Li et al., 2019). Such *Taq*Man‐based triplex real‐time PCR methods for poultry authentication including chicken, duck, goose, and endogenous control, albeit appropriate, have not been developed so far.

The goal of this study was to develop a triplex real‐time PCR method for the simultaneous identification of chicken, duck, and goose DNA in meat. More importantly, an endogenous control was designed to be compatible with chicken, duck, and goose probes, and was amplified with poultry‐specific probes in order to eliminate false negative results, which is known to be a serious limitation to the legal impartiality of the report of authentication test.

## 2 MATERIALS AND METHODS

### 2.1 Preparation of meat samples and DNA extraction

Raw meat and products from chicken, duck, goose, quail, pigeon, cattle, buffalo, yak, sheep, goat, pig, horse, donkey, camel, and rabbit were obtained from agricultural market in Xilinhot, China. The meat samples were chopped into small pieces and stored at −80°C to prevent degradation of DNA.

The genomic DNA from meat samples was extracted by Takara MiniBEST Universal Genomic DNA Extraction Kit (TaKaRa, Dalian, China) according to manufacturer's protocol. The purity and concentration of extracted DNA were determined based on absorbance at *A*_260_/*A*_280_ by Nanodrop2000 (Thermo Fisher Scientific).

### 2.2 Development of primers and probes for the triplex real‐time PCR

**FIGURE 1:**
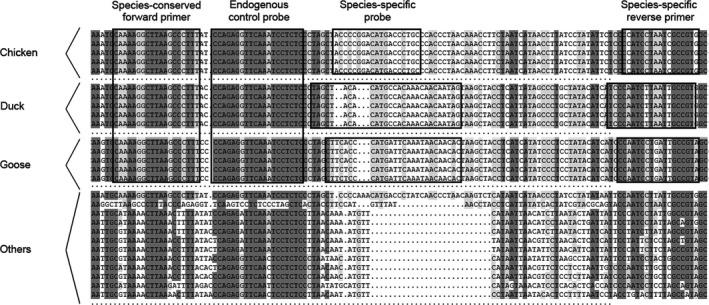
Design of primers and probes for the improved triplex real‐time PCR for synchronous identification of DNA from chicken, duck, and goose meat. The forward primer and endogenous control probe are species‐conserved for chicken, duck, and goose mitochondrial DNA, and the reverse primer and poultry probe are species‐specific

Triplex real‐time PCR involves three amplification reactions, which require three independent primer pairs and three probes. Functionally, three different probes anneal with the corresponding species‐specific DNA and participate in the PCR reaction with primers. From our previous experience, we purpose that a species‐conserved forward primer, a species‐specific reverse primer, and three species‐specific probes are more compatible and stable than three independent primer pairs in the triplex real‐time PCR. In this study, after alignment of mitochondrial DNA from different species (chicken, duck, goose, quail, pigeon, cattle, buffalo, yak, sheep, goat, pig, horse, donkey, camel, and rabbit), the species‐conserved forward primer, the species‐specific reverse primer, and the species‐specific probes were designed to specifically target the mitochondrial gene sequences of chicken, duck, and goose, respectively (Figure 1). Additionally, a species‐conserved probe for endogenous control was designed to monitor the amplification reaction, and eliminate false negative detection (Figure 1). The primers and probes for our triplex real‐time PCR were designed to anneal with a limited DNA length between 100 and 150 nucleotides. Additionally, different fluorescent reporters, such as 6‐carboxyfluorescein (FAM), hexacholoro‐6‐corboxyfluorescein (HEX), and carboxy‐X‐rhodamine (ROX), were introduced to label chicken, duck, goose, and the endogenous control probe, respectively. All oligonucleotides were synthesized and purified using HPLC by Ruibiotech Company, and showed in Table 1.

**TABLE 1:** Sequences of the designed primers and probes

Primer/probe	Sequence 5′–3′	Labeling
Forward primer	CAAAAGGCTTAAGCCCTTT	None
Chicken reverse primer	CACGGCGATTAGGATGG	None
Duck reverse primer	ACGGCAATTAAGATTGGGA	None
Goose reverse primer	TACGGCAATCAGGATTGG	None
Chicken probe	ACCCCGGACATGACCCTGC	FAM‐TAMARA
Duck probe	TAGCTACACATGCCACAAACAACAATAG	HEX‐TAMARA
Goose probe	CTTC(A/T)CCCATGATTCAAATAACAACAC	ROX‐BHQ2
Endogenous control probe	CCAGAGGTTCAAATCCTCTC	ROX‐MGB

### 2.3 Specificity assay

DNA from raw meat (chicken, duck, goose, quail, pigeon, cattle, buffalo, yak, sheep, goat, pig, horse, donkey, camel, and rabbit) and processed meat products (chicken sausage, spiced duck wing, goose jerky, chicken pork sausage, chicken beef sausage, beef jerky, mutton jerky, dried horse meat, and dried donkey meat) were used to confirm the specificity of the triplex real‐time PCR, and the results were verified by three replicates. The triplex real‐time PCR systems consisted of 10 μl of TransStart probe qPCR SuperMix (Tansgen), each of 1 μl forward primer (10 μM), 1 μl reverse primer (including reverse primers of chicken, duck, and goose), 0.5 μl of chicken probe (10 μM), 0.5 μl of duck probe (10 μM), 0.5 μl of goose probe or control probe (10 μM), 1 μl of DNA template (100 ng/μl), and distilled deionized water (Transgen) for a total volume of 20 μL. The amplification was performed using initial denaturation step at 94°C for 30 s, followed by 40 cycles of denaturation at 94°C for 5 s, and annealing and extension at 60°C for 34 s. (ABI 7300plus, Applied Biosystems).

### 2.4 Sensitivity and authentication assay

The sensitivity assay of the triplex real‐time PCR was evaluated by the limit of detection (LOD). To determine the LOD, total DNA of the target species (chicken, duck, and goose) was diluted using 10‐fold and twofold serial dilutions (100, 10, 1, 0.1, 0.01, 0.005, 0.0025, 0.001, 0.0005, 0.00025, 0.0001, and 0.00001 ng/μl) (Table 3). Twenty replicates for each dilution were used for the evaluation of LOD of the triplex real‐time PCR, and the results were analyzed as inferred from Probit analysis (Finney, 1971).

Ternary meat mixtures containing chicken, duck, and goose were prepared to evaluate the authentication ability of the triplex real‐time PCR (Table 4). First, the percentages of chicken in the mixtures were 0.1%, 1%, 10%, and 30% (w/w), the percentages of duck in the mixtures were 0.1%, 1%, 10%, and 30% (w/w), and the corresponding percentages of goose in the mixtures were 99.8%, 98%, 80%, and 40% (w/w). Second, the percentages of chicken meat in the mixtures were 0.1%, 1%, 10%, and 30% (w/w), the percentages of duck in the mixtures were 99.8%, 98%, 80%, and 40% (w/w), and the corresponding percentages of goose meat in the mixtures were 0.1%, 1%, 10%, and 30% (w/w). Last, the percentages of duck in the mixtures were 0.1%, 1%, 10%, and 30% (w/w), the percentages of chicken in the mixtures were 99.8%, 98%, 80%, and 40% (w/w), and the corresponding percentages of goose meat in the mixtures were 0.1%, 1%, 10%, and 30% (w/w). The DNA extracted from these meat mixtures was utilized as the template for the triplex real‐time PCR. In addition, binary meat mixtures containing two meats from chicken, duck, and goose were utilized to evaluate the authentication of the triplex real‐time PCR containing an endogenous control, and the percentages of chicken, duck, or goose in the mixtures were 0.1%, 1%, 10%, 30%, 70%, 90%, 99%, and 99.9% (w/w), and the corresponding percentages of meat in the mixtures were 99.9%, 99%, 90%, 70%, 30%, 10%, 1%, and 0.1% (w/w). Twenty replicates for each dilution were used for the evaluation of LOD of triplex real‐time PCR, and the results were analyzed as inferred from Probit analysis (Finney, 1971).

## 3 RESULTS AND DISCUSSION

### 3.1 Specificity evaluation of the triplex real‐time PCR reaction

**FIGURE 2:**
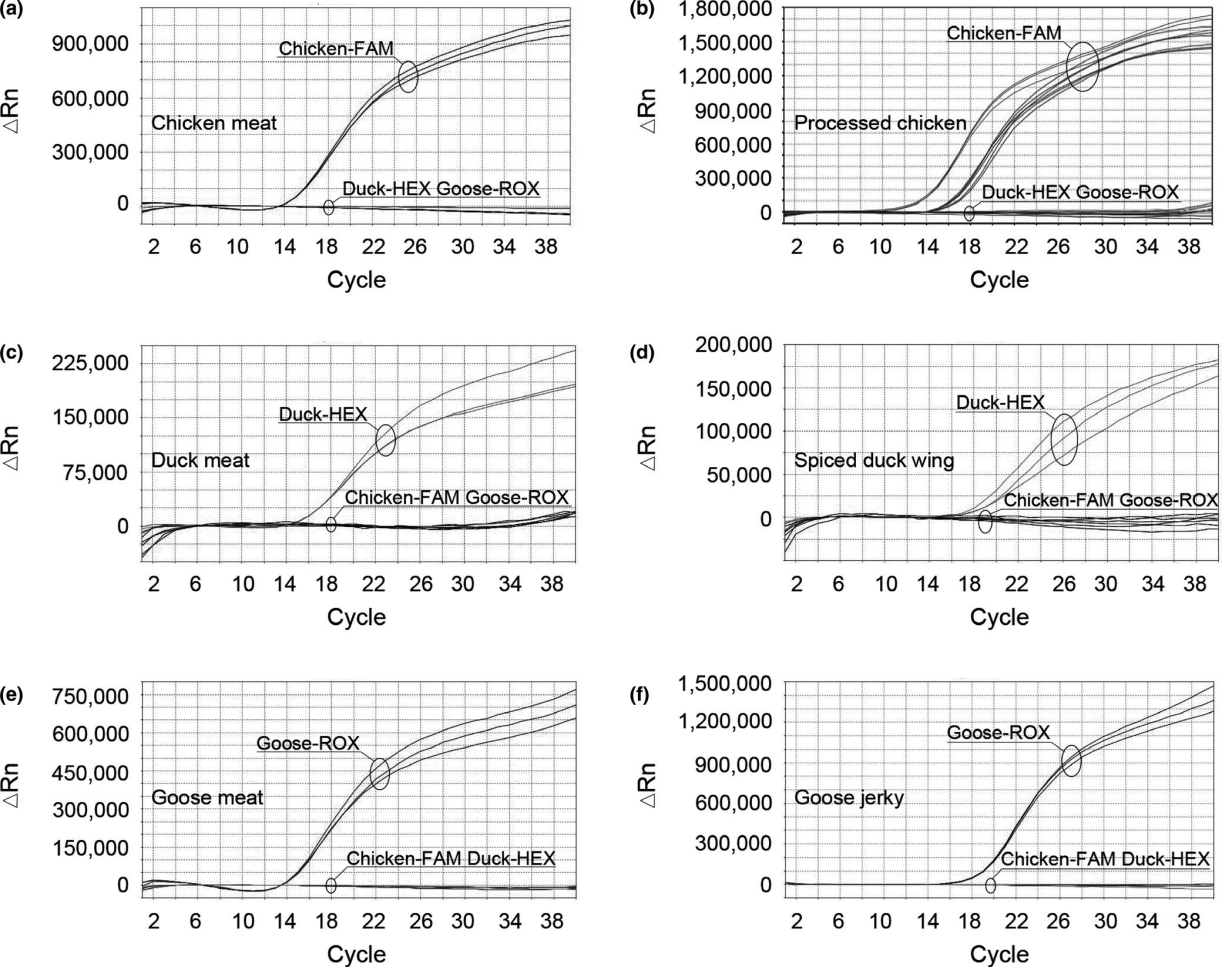
Triplex real‐time PCR amplification curves for specificity evaluation. The results were confirmed by 3 replicates. ΔRn = change in normalized reported values

The specificity of the triplex real‐time PCR assay was determined using the designed probes to identify the corresponding DNA from chicken, duck, and goose. As shown in Figure 2, the amplification curves of chicken‐FAM were specifically observed in chicken meat (Figure 2a) and processed chicken (chicken sausage, chicken pork sausage, and chicken beef sausage; Figure 2b). The amplification curves of duck‐HEX were specifically observed in duck meat (Figure 2c) and spiced duck wing (Figure 2d), and the amplification curves of goose‐ROX were specifically observed in goose meat (Figure 2e) and goose jerky (Figure 2f). As shown in Table 2, the cycle threshold (Ct) values (average ± *SD*) of the triplex real‐time PCR method with chicken, duck, and goose probes were consistent with the amplification curves. These results indicate that the chicken, duck, and goose probes can simultaneously identify their target DNA, and that the triplex real‐time PCR method is specific in the detection of distinct poultry meats.

**TABLE 2:** Specificity assay of the triplex real‐time PCR technique

Samples	Chicken–duck–goose group			Chicken–duck–control group		
	Chicken‐FAM	Duck‐HEX	Goose‐ROX	Chicken‐FAM	Duck‐HEX	Control‐ROX
Chicken	13.69 ± 0.02^a^	0.00	0.00	15.47 ± 1.59	0.00	13.56 ± 0.47
Duck	0.00	13.82 ± 0.38	0.00	0.00	24.02 ± 0.15	19.76 ± 0.92
Goose	0.00	0.00	13.65 ± 0.34	0.00	0.00	13.61 ± 0.01
Quail	0.00	0.00	0.00	0.00	0.00	N/A^b^
Pigeon	0.00	0.00	0.00	0.00	0.00	N/A
Cattle	0.00	0.00	0.00	0.00	0.00	N/A
Buffalo	0.00	0.00	0.00	0.00	0.00	N/A
Yak	0.00	0.00	0.00	0.00	0.00	N/A
Sheep	0.00	0.00	0.00	0.00	0.00	N/A
Goat	0.00	0.00	0.00	0.00	0.00	N/A
Pig	0.00	0.00	0.00	0.00	0.00	N/A
Horse	0.00	0.00	0.00	0.00	0.00	N/A
Donkey	0.00	0.00	0.00	0.00	0.00	N/A
Camel	0.00	0.00	0.00	0.00	0.00	N/A
Rabbit	0.00	0.00	0.00	0.00	0.00	N/A
Chicken sausage	14.19 ± 0.07	0.00	0.00	13.56 ± 0.01	0.00	13.61 ± 0.02
Chicken pork sausage	13.82 ± 0.03	0.00	0.00	14.33 ± 0.16	0.00	13.58 ± 0.05
Chicken beef sausage	13.84 ± 0.03	0.00	0.00	15.48 ± 0.21	0.00	13.69 ± 0.15
Spiced duck wing	0.00	13.50 ± 0.21	0.00	0.00	24.02 ± 0.15	19.76 ± 0.92
Goose jerky	0.00	0.00	13.54 ± 0.01	0.00	0.00	13.61 ± 0.01
Beef jerky	0.00	0.00	0.00	0.00	0.00	N/A
Mutton jerky	0.00	0.00	0.00	0.00	0.00	N/A
Dried horse meat	0.00	0.00	0.00	0.00	0.00	N/A
Dried donkey meat	0.00	0.00	0.00	0.00	0.00	N/A

^a^ Ct value: Average ± *SD* from three replicates.

^b^ Not applicable.

Although efforts have been made to develop efficient methods for the detection of poultry contamination in meat (Amaral et al., 2015; Furutani et al., 2017; Hou et al., 2015; Kesmen et al., 2012; Martin et al., 2007; Pegels et al., 2012; Thanakiatkrai et al., 2019), only two studies reported multiplex PCR techniques containing an end point PCR method (Hou et al., 2015) and a *Taq*Man‐based multiplex real‐time method (Köppel et al., 2013). However, the protocol based on multiplex PCR was shown to be less efficient, more time‐consuming, and more laborious than our approach, which does not require electrophoresis of the amplified DNA (Hou et al., 2015).

### 3.2 Sensitivity evaluation of the triplex real‐time PCR reaction

**FIGURE 3:**
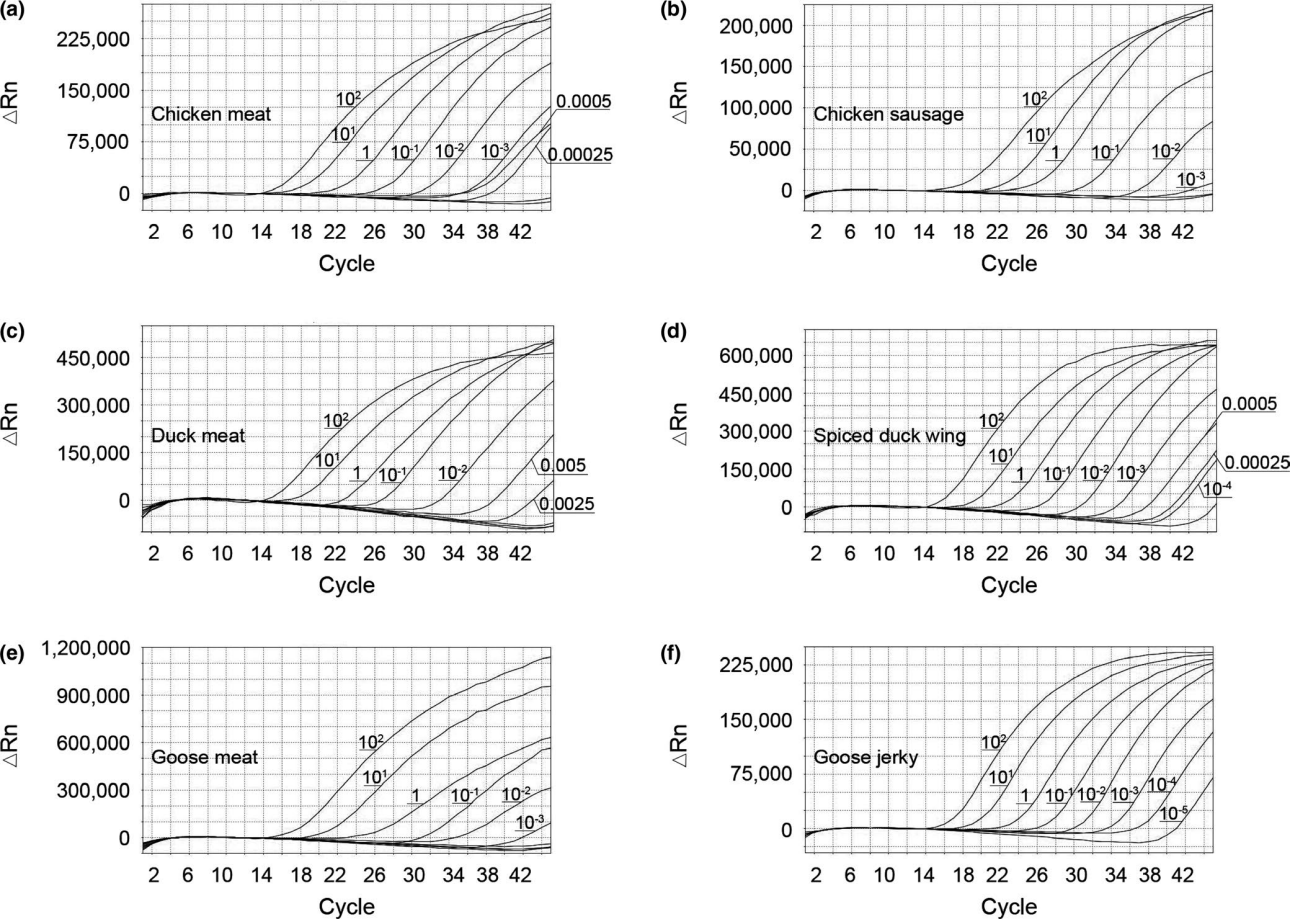
Triplex real‐time PCR amplification curves for sensitivity evaluation. The results were confirmed by 20 replicates. ΔRn = change in normalized reported values

According to the parameters required for the development and validation of the triplex real‐time PCR method, the LOD is defined as the lowest concentration of the analyte with positive amplification at least 95% of independent amplification reactions (Bustin et al., 2009; Marchesi et al., 2015). The LODs of chicken meat and chicken sausage, in the reaction with the chicken‐FAM probe, were 0.00025 ng (Figure 3a) and 0.001 ng (Figure 3b), respectively. For the duck meat and spiced duck wing with the duck‐HEX probe, the obtained LODs were 0.0025 ng (Figure 3c) and 0.0001 ng (Figure 3d), respectively. Finally, the LODs of goose meat and goose jerky in the reaction with the goose‐ROX probe were of 0.001 ng (Figure 3e) and 0.00001 ng (Figure 3f), respectively. The Ct values (average ± *SD*) of the triplex real‐time PCR increased with increasing dilution of meat DNA (twenty replicates per sample; Table 3). The above results suggested that the LOD was different between meat samples. We speculated that the integrity of DNA influences the LOD of the real‐time PCR reaction. The LODs observed in this study were lower than in previously reported multiplex PCR method (Hou et al., 2015) and were similar to other real‐time PCR approaches (Guo et al., 2018; Kesmen et al., 2012). These results show that our newly developed real‐time PCR is sensitive in the detection of poultry DNA in meat products.

**TABLE 3:** Sensitivity assay of the triplex real‐time PCR technique

Sample	Template (ng)	Ct value^a^	Number of positive replicates	Confidence limit (%)
Chicken	100	11.54 ± 0.35	20/20	100
	10	15.21 ± 0.29	20/20	100
	1	20.10 ± 0.66	20/20	100
	0.1	24.76 ± 1.76	20/20	100
	0.01	31.13 ± 1.13	20/20	100
	0.001	36.06 ± 0.58	20/20	100
	0.0005	37.41 ± 0.39	20/20	100
	0.00025	38.34 ± 0.59	20/20	100
	0.0001	42.29 ± 0.60	7/20	35
	0.00001	0.00	0/20	0
Chicken sausage	100	14.35 ± 0.37	20/20	100
	10	18.00 ± 0.96	20/20	100
	1	23.68 ± 0.92	20/20	100
	0.1	29.65 ± 1.06	20/20	100
	0.01	35.88 ± 1.86	20/20	100
	0.001	39.67 ± 0.92	20/20	100
	0.0001	42.76 ± 0.00	1/20	5
	0.00001	0.00	0/20	0
Duck meat	100	17.20 ± 0.76	20/20	100
	10	22.64 ± 1.07	20/20	100
	1	26.67 ± 0.53	20/20	100
	0.1	31.27 ± 0.99	20/20	100
	0.01	35.72 ± 1.12	20/20	100
	0.005	36.85 ± 0.87	20/20	100
	0.0025	37.98 ± 0.96	20/20	100
	0.001	41.38 ± 0.46	15/20	75
	0.0001	40.89 ± 1.43	17/20	85
	0.00001	41.03 ± 0.51	3/20	15
Spiced duck wing	100	14.25 ± 0.73	20/20	100
	10	16.92 ± 0.38	20/20	100
	1	21.55 ± 0.43	20/20	100
	0.1	26.05 ± 0.41	20/20	100
	0.01	30.25 ± 0.33	20/20	100
	0.001	34.62 ± 0.36	20/20	100
	0.0005	38.31 ± 0.56	20/20	100
	0.00025	40.65 ± 0.92	20/20	100
	0.0001	41.60 ± 0.89	20/20	100
	0.00001	41.81 ± 0.71	12/20	60
Goose meat	100	13.72 ± 0.04	20/20	100
	10	16.56 ± 0.84	20/20	100
	1	21.20 ± 0.85	20/20	100
	0.1	26.91 ± 0.71	20/20	100
	0.01	31.87 ± 0.60	20/20	100
	0.001	37.14 ± 0.57	20/20	100
	0.0005	39.69 ± 0.90	15/20	75
	0.00025	41.54 ± 0.59	14/20	70
	0.0001	41.85 ± 0.57	12/20	60
	0.00001	0.00	0/20	0
Goose jerky	100	14.54 ± 0.36	20/20	100
	10	17.42 ± 0.84	20/20	100
	1	21.88 ± 0.81	20/20	100
	0.1	26.13 ± 0.90	20/20	100
	0.01	30.68 ± 0.86	20/20	100
	0.001	34.83 ± 1.09	20/20	100
	0.0001	37.81 ± 1.30	20/20	100
	0.00001	39.98 ± 0.84	20/20	100

^a^ Average ± *SD* from 20 replicates.

**FIGURE 4:**
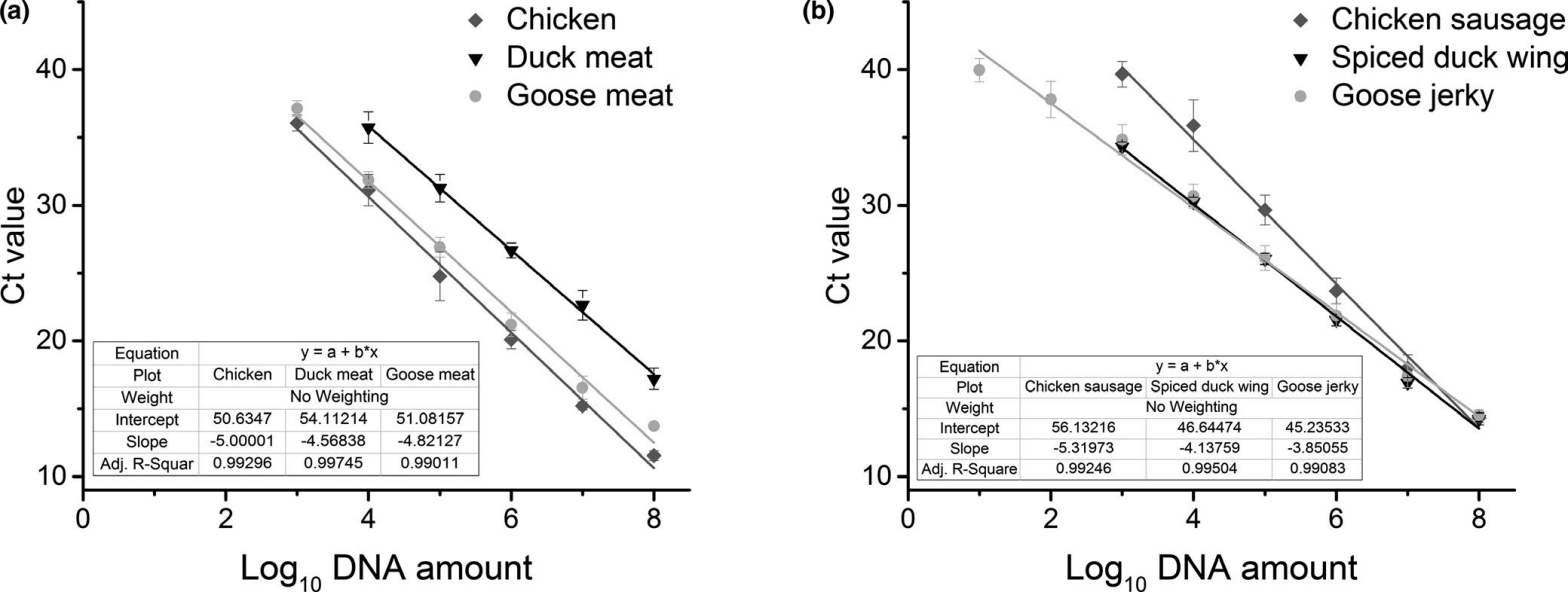
Quantification assays in poultry: chicken, duck meat, and goose meat (a) and chicken sausage, spiced duck wing, and goose jerky (b)

To determine the linearity of the triplex real‐time PCR assay, DNA from meat products was serially diluted and used as template. Calibration curves were constructed by plotting the resulting Ct values against the logarithm of DNA concentrations. The calibration curves of chicken, duck meat, goose meat, chicken sausage, spiced duck wing, and goose jerky were shown in Figure 4. This shows a significant linear relationship between the Ct values and the logarithm of the DNA concentrations. According to the general guidelines described by European Network of GMO Laboratories (Marchesi et al., 2015), the parameter of the triplex real‐time PCR methods to comply with the acceptance criteria established for this type of assay is a correlation coefficient (*R* ^2^) above 0.98. Thus, our results show that our method is in accordance with the established guidelines for the quantitative determination of the raw and processed poultry.

### 3.3 Authentication evaluation of the triplex real‐time PCR reaction

**FIGURE 5:**
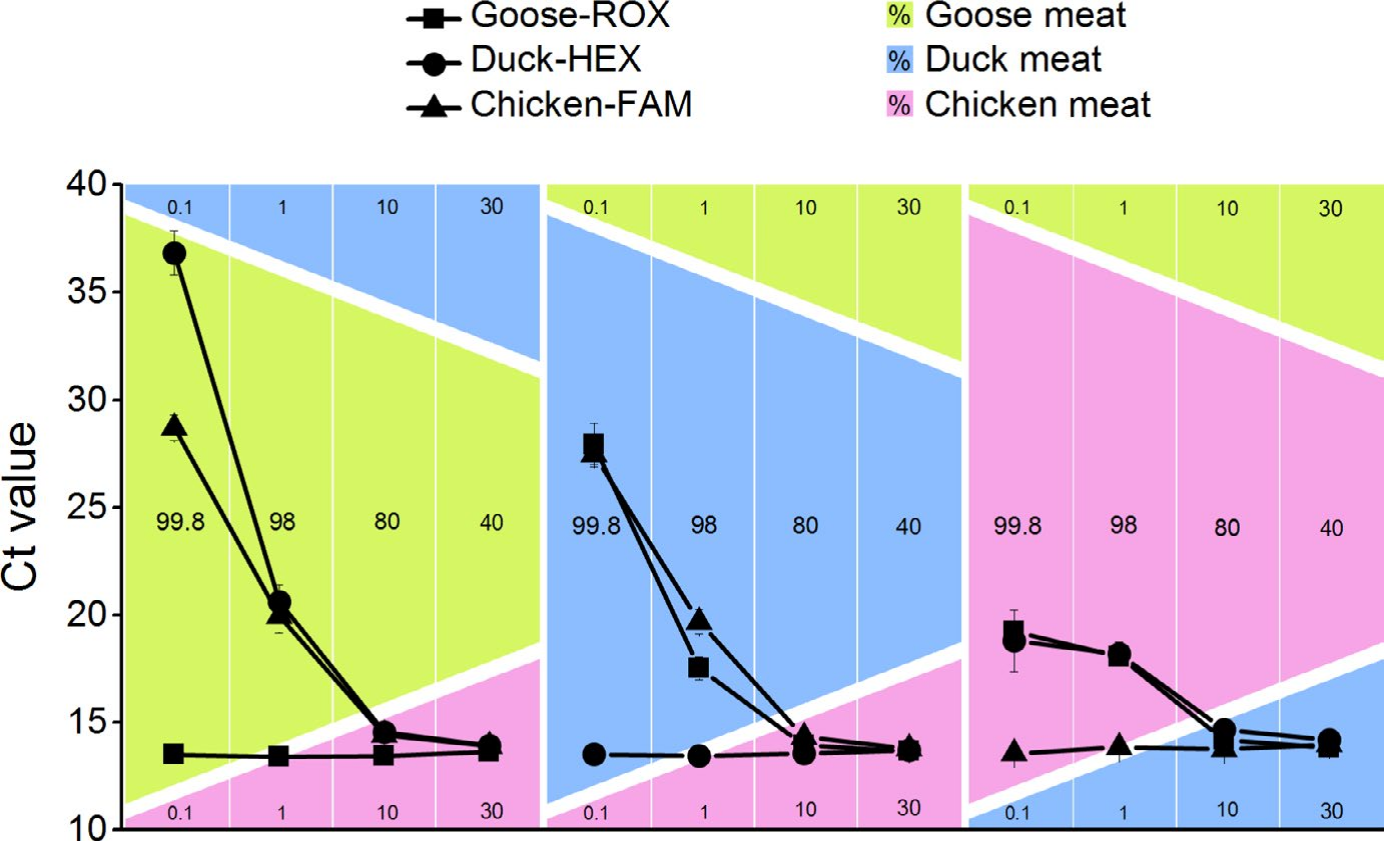
Validation assays in ternary meat mixtures. The results were confirmed by 20 replicates

In order to simulate the poultry adulteration practice and to validate the simultaneous triplex real‐time PCR method for authentication, the method was utilized to detect chicken, duck, and goose DNA in the ternary meat mixtures of chicken, duck, and goose. As shown in Figure 5 and Table 4, the three target poultry species were detected in the ternary meat mixtures, at levels as low as 0.1% of total meat weight. Moreover, the triplex real‐time PCR method was successfully employed for simultaneous detection of two target species in the three types of ternary meat mixtures, at levels as low as 0.1% of total meat weight (Figure 5). These results revealed that the triplex real‐time PCR method is a sensitive and specific approach for the rapid and simultaneous identification of minimal percentages of two target species in meat mixtures. It is frequently observed that the inclusion of poultry products in other meats, for economic profit, corresponds to over 10% of total meat weight. Thus, we purpose that our method might be useful in the market supervision of meat adulteration.

**TABLE 4:** Authentication assay of the triplex real‐time PCR in the ternary and binary meat mixtures

Mass (%)			Ct value^a^			
Chicken	Duck meat	Goose meat	Chicken‐FAM	Duck‐HEX	Goose‐R.H.F^c^	Control‐ROX
0.1	0.1	99.8	28.71 ± 0.57	36.84 ± 1.01	13.51 ± 0.23	N/A^b^
1	1	98	19.95 ± 0.77	20.61 ± 0.78	13.41 ± 0.17	N/A
10	10	80	14.42 ± 0.19	14.55 ± 0.18	13.45 ± 0.02	N/A
30	30	40	13.91 ± 0.06	13.93 ± 0.09	13.65 ± 0.04	N/A
0.1	99.8	0.1	27.47 ± 0.46	13.53 ± 0.03	27.91 ± 0.98	N/A
1	98	1	19.69 ± 0.56	13.45 ± 0.04	17.52 ± 0.51	N/A
10	80	10	14.33 ± 0.23	13.55 ± 0.06	13.94 ± 0.10	N/A
30	40	30	13.78 ± 0.03	13.82 ± 0.04	13.66 ± 0.03	N/A
99.8	0.1	0.1	13.56 ± 0.01	18.80 ± 1.40	19.25 ± 0.33	N/A
98	1	1	13.85 ± 0.03	18.19 ± 0.53	18.06 ± 0.32	N/A
80	10	10	13.75 ± 0.03	14.67 ± 0.18	14.18 ± 0.10	N/A
40	30	30	13.99 ± 0.07	14.19 ± 0.07	13.83 ± 0.04	N/A
0.1	99.9	N/A	22.67 ± 0.93	13.52 ± 0.14	N/A	13.43 ± 0.06
1	99	N/A	16.82 ± 0.41	13.59 ± 0.12	N/A	13.53 ± 0.05
10	90	N/A	14.89 ± 0.76	13.66 ± 0.17	N/A	13.49 ± 0.06
30	70	N/A	13.94 ± 0.38	13.81 ± 0.23	N/A	13.56 ± 0.02
70	30	N/A	13.70 ± 0.21	14.10 ± 0.37	N/A	13.60 ± 0.03
90	10	N/A	13.82 ± 0.25	14.48 ± 0.46	N/A	13.61 ± 0.13
99	1	N/A	13.73 ± 0.13	15.23 ± 0.48	N/A	13.60 ± 0.10
99.9	0.1	N/A	13.78 ± 0.09	17.12 ± 0.86	N/A	13.55 ± 0.10
0.1	N/A	99.9	34.65 ± 1.08	N/A	13.80 ± 0.10	13.58 ± 0.01
1	N/A	99	18.85 ± 0.87	N/A	13.58 ± 0.06	13.44 ± 0.02
10	N/A	90	14.65 ± 0.41	N/A	13.72 ± 0.11	13.54 ± 0.02
30	N/A	70	13.84 ± 0.08	N/A	13.73 ± 0.08	13.55 ± 0.02
70	N/A	30	13.65 ± 0.03	N/A	14.03 ± 0.15	13.62 ± 0.02
90	N/A	10	13.64 ± 0.03	N/A	14.94 ± 0.48	13.65 ± 0.01
99	N/A	1	13.56 ± 0.03	N/A	16.30 ± 0.67	13.57 ± 0.02
99.9	N/A	0.1	13.66 ± 0.02	N/A	22.94 ± 1.49	13.63 ± 0.02
N/A	0.1	99.9	N/A	39.67 ± 0.93	13.46 ± 0.03	13.45 ± 0.02
N/A	1	99	N/A	25.78 ± 1.18	13.85 ± 0.20	13.62 ± 0.02
N/A	10	90	N/A	14.69 ± 0.32	13.38 ± 0.06	13.42 ± 0.02
N/A	30	70	N/A	13.94 ± 0.09	13.66 ± 0.08	13.56 ± 0.01
N/A	70	30	N/A	13.63 ± 0.06	14.19 ± 0.25	13.57 ± 0.02
N/A	90	10	N/A	13.45 ± 0.03	16.05 ± 0.51	13.47 ± 0.02
N/A	99	1	N/A	13.48 ± 0.01	26.08 ± 0.49	13.50 ± 0.02
N/A	99.9	0.1	N/A	13.46 ± 0.01	0.00	13.51 ± 0.02

^a^ Ct value: Average ± *SD* from 20 replicates.

^b^ Not applicable.

^c^ The goose probe was labeled with ROX, HEX, or FAM fluorescence, respectively.

PCR‐based methods have been previously used to identify as little as 0.1% pork content (Karabasanavar et al., 2014), 0.1% dog meat (Rahman et al., 2014), and 0.1% chicken content in meat mixtures (Furutani et al., 2017). Yet, to our knowledge, no PCR‐based methods for the simultaneous identification of two species, maintaining the same detection limit, have been developed so far. Previously developed triplex real‐time PCR methods for the simultaneous identification of mare and cow, as well as goat and cow, in milk, showed less sensitivity (1%–10%) (Guo et al., 2018, 2019). The triplex real‐time PCR for mare, goat, and cow milk used a species‐conserved primer pair. Conversely, we hypothesize that the species‐conserved forward primer ensured the performance of the triplex real‐time PCR, while the species‐specific reverse primer increased the authentication ability of the method in this study.

### 3.4 Endogenous control validation

**FIGURE 6:**
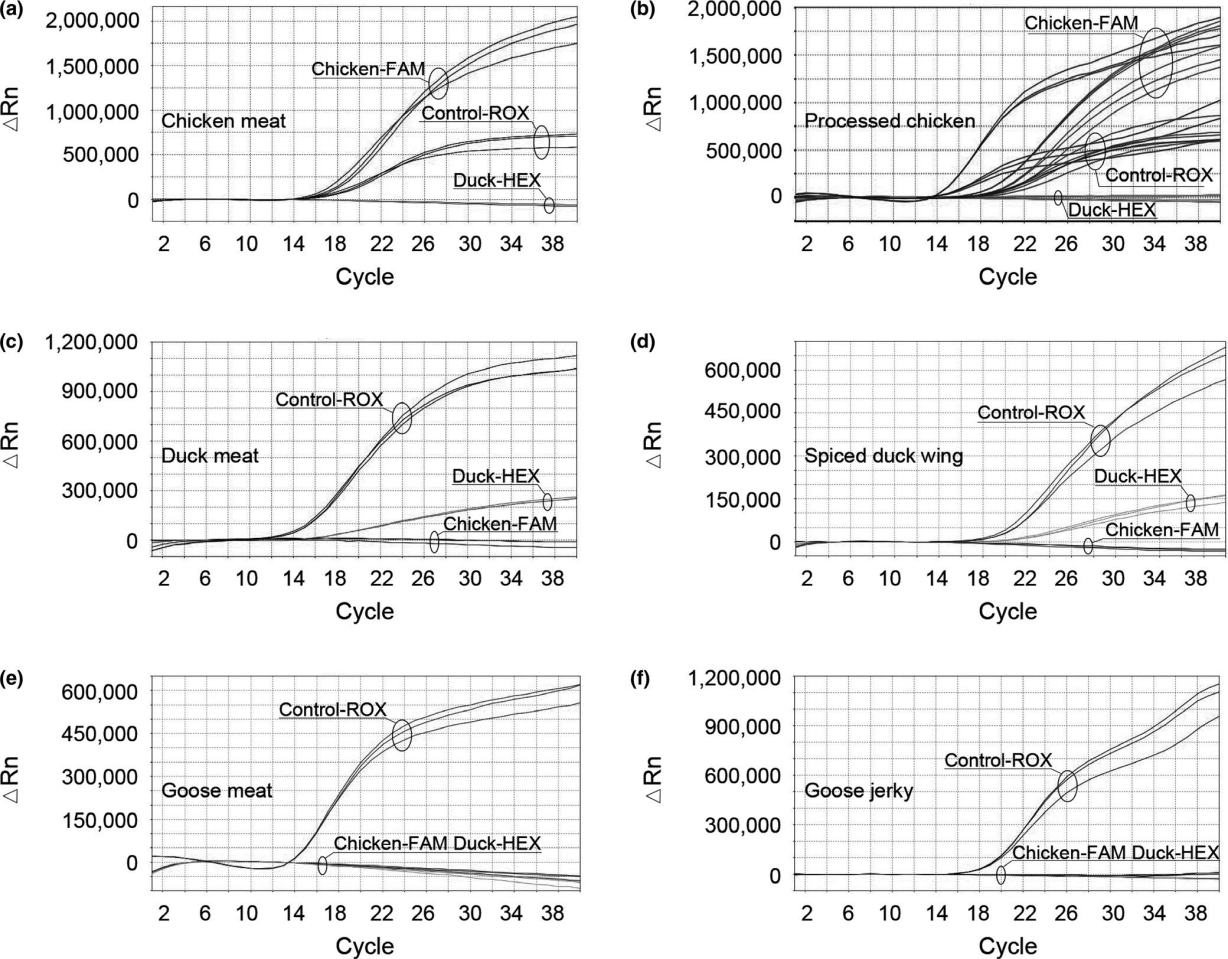
Triplex real‐time PCR amplification curves for specificity evaluation, with endogenous control probe. The results were confirmed by 3 replicates. ΔRn = change in normalized reported values

We designed an endogenous probe to be amplified with chicken‐, duck‐, and goose‐specific probes, to avoid false negative results. The specificity of the triplex real‐time PCR containing Chicken‐FAM, Duck‐HEX, and Control‐ROX was validated using chicken, duck, and goose meats. As expected from our previous findings, the amplification curves of chicken‐FAM were specifically observed for chicken meat (Figure 6a) and processed chicken (Figure 6b), and the amplification curves of duck‐HEX were specifically observed for duck meat (Figure 6c) and spiced duck wing (Figure 6d). More importantly, the amplification curves of Control‐ROX were detected in all poultry samples (Figure 6). Although the assembled multiplex real‐time PCR has been developed to detect simultaneously chicken, duck, and goose (Köppel et al., 2013), our results suggest that the triplex real‐time PCR with the amplification of endogenous control in this study might be more effective and accurate, due to the elimination of false negative results.

**FIGURE 7:**
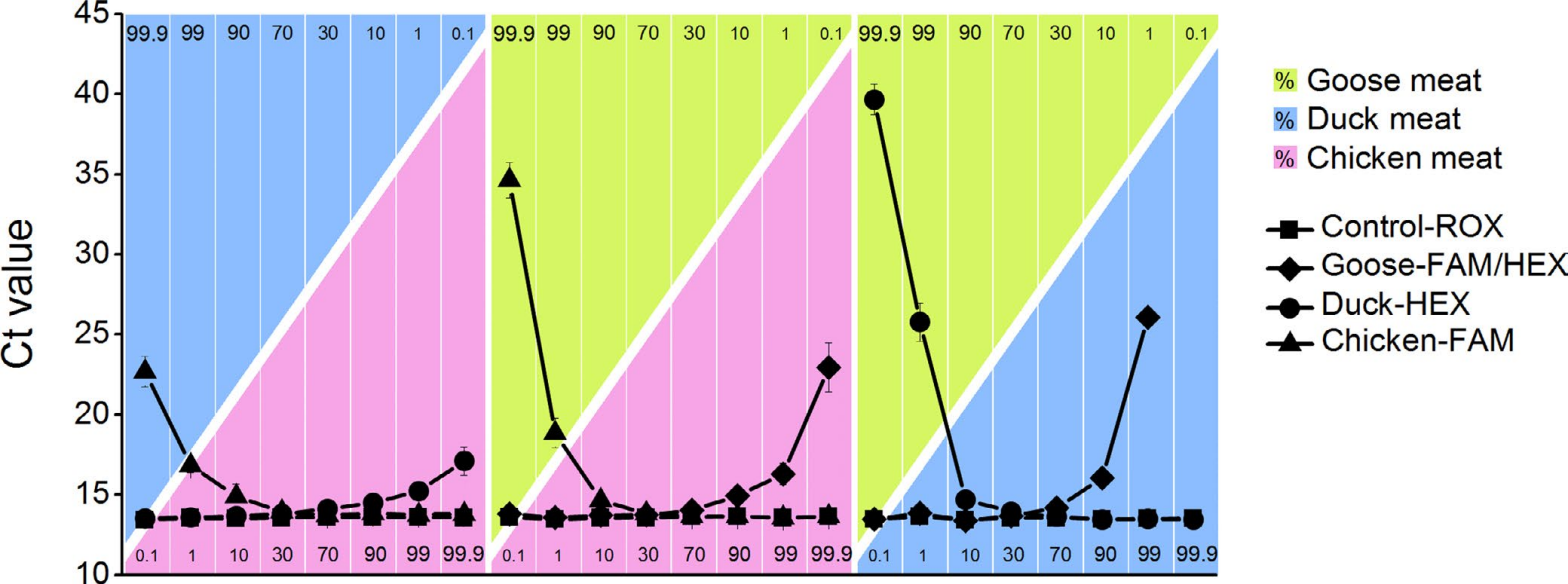
Validation assays in binary meat mixtures. The results were confirmed by 20 replicates

The three triplex real‐time PCR reactions (Chicken‐FAM, Duck‐HEX, and Control‐ROX; Chicken‐FAM, Goose‐HEX, and Control‐ROX; Goose‐FAM, Duck‐HEX, and Control‐ROX) were validated in the corresponding binary meat mixtures. As shown in Figure 7 and Table 4, the three target species (chicken, duck, and goose) were identified at low concentration (0.1%), and the endogenous control was successfully amplified with species‐specific probes in the three types of the binary meat mixtures. These results demonstrate that the triplex real‐time PCR method is sensitive and specific for rapid identification of very low percentages of poultry in meat mixtures.

In this study, we used ABI 7300plus to perform the triplex real‐time PCR. This instrument can simultaneously detect three different fluorescent probes. Therefore, the three triplex real‐time PCR system was assembled from the four probes developed in this study. Moreover, these probes can be combined into a quadruplex real‐time PCR method, allowing for the simultaneous detection of meat origins and the amplification of the endogenous control.

## 4 CONCLUSIONS

To study the adulteration in poultry and increase the efficiency and sensitivity of real‐time PCR, we developed the triplex real‐time PCR for the simultaneous identification of chicken, duck, and goose DNA. The improved triplex real‐time PCR containing the species‐conserved forward primer, the species‐specific reverse primer, and three species‐specific probes is more efficient and economical than previously reported methods using conventional real‐time PCR. Furthermore, an endogenous probe has been designed to be amplified with poultry‐specific probes in order to avoid false negative results. The limits of detection of chicken, duck, and goose in the improved triplex real‐time PCR were 0.001–0.00025 ng, 0.0025–0.0001 ng, and 0.001–0.00001 ng, respectively. In addition, 0.1% poultry adulteration can be steadily validated in the simulation of adulteration. In conclusion, our triplex real‐time PCR method with an endogenous control shows higher specificity, sensitivity, and efficiency in the synchronous identification of chicken, duck, and goose DNA in meat products.

## CONFLICT OF INTEREST

All authors declare no conflict of interest.

## ETHICAL APPROVAL

This study does not involve any human or animal testing.

## Data Availability

The data that support the findings of this study are available from the corresponding author upon reasonable request.
